# Supplementary material for: Mega-Dose Vitamin C Ameliorates Nonalcoholic Fatty Liver Disease in a Mouse Fast-Food Diet Model
Source: Nutrients. 2022 May 25;14(11):2195. doi: 10.3390/nu14112195 (PMC9182669; doi:10.3390/nu14112195)
Supplement: Supplementary file 1 [file nutrients-14-02195-s001.zip › Revised supplementary figure.pdf]

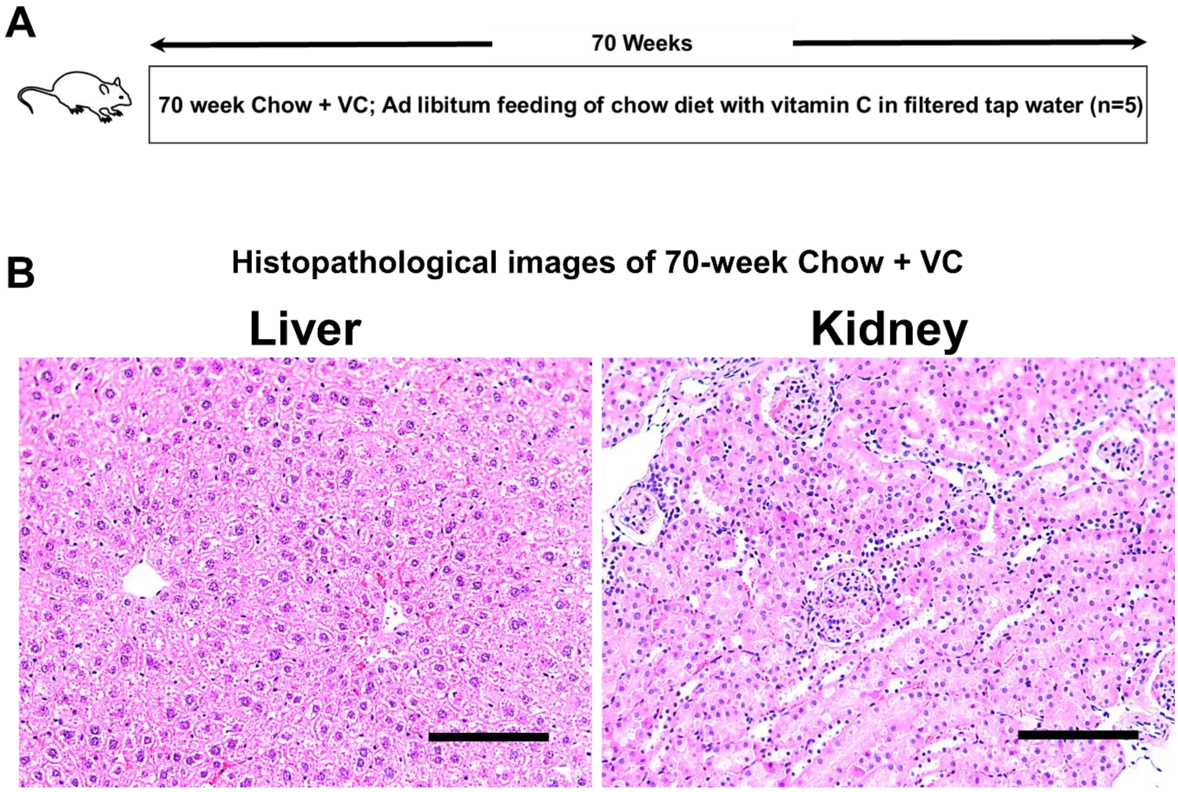

Supplementary Figure S1. The histopathological images of the mice treated for 70 weeks with vitamin C. **(A)**: Schematic diagram of the experimental protocol used to confirm the safety of long-term mega-dose vitamin C treatment. **(B)**: Representative images of H&E staining of liver and kidney. Scale bars = 100 μm (B). Original magnification, X 200 (B).
